# Computed tomography-based radiomic markers are independent prognosticators of survival in advanced laryngeal cancer: a pilot study

**DOI:** 10.1017/S0022215123002372

**Published:** 2024-06

**Authors:** Amarkumar Dhirajlal Rajgor, Christopher Kui, Andrew McQueen, Josh Cowley, Colin Gillespie, Aileen Mill, Stephen Rushton, Boguslaw Obara, Theophile Bigirumurame, Khaled Kallas, James O'Hara, Eric Aboagye, David Winston Hamilton

**Affiliations:** 1 Newcastle University, Newcastle-Upon-Tyne, UK; 2 Population Health Sciences Institute, Newcastle University, Newcastle-Upon-Tyne, UK; 3 Newcastle-Upon-Tyne Hospitals NHS Foundation Trust, Freeman Hospital, Freeman Road, Newcastle-Upon-Tyne, UK; 4 Imperial College London Cancer Imaging Centre, Department of Surgery & Cancer, Hammersmith Hospital, London, UK

**Keywords:** Laryngeal neoplasms, biomarkers, prognosis

## Abstract

**Objective:**

Advanced laryngeal cancers are clinically complex; there is a paucity of modern decision-making models to guide tumour-specific management. This pilot study aims to identify computed tomography-based radiomic features that may predict survival and enhance prognostication.

**Methods:**

Pre-biopsy, contrast-enhanced computed tomography scans were assembled from a retrospective cohort (*n* = 72) with advanced laryngeal cancers (T3 and T4). The LIFEx software was used for radiomic feature extraction. Two features: shape compacity (irregularity of tumour volume) and grey-level zone length matrix – grey-level non-uniformity (tumour heterogeneity) were selected via least absolute shrinkage and selection operator-based Cox regression and explored for prognostic potential.

**Results:**

A greater shape compacity (hazard ratio 2.89) and grey-level zone length matrix – grey-level non-uniformity (hazard ratio 1.64) were significantly associated with worse 5-year disease-specific survival (*p* < 0.05). Cox regression models yielded a superior C-index when incorporating radiomic features (0.759) versus clinicopathological variables alone (0.655).

**Conclusions:**

Two radiomic features were identified as independent prognostic biomarkers. A multi-centre prospective study is necessary for further exploration. Integrated radiomic models may refine the treatment of advanced laryngeal cancers.

## Introduction

Laryngeal cancer affects approximately 2400 individuals annually in the UK, comprising a large proportion (10–20 per cent) of all head and neck cancers.^1,2^ Presenting symptoms include hoarse voice, pain or progressive dysphagia.^2^ Assessment requires endoscopic visualisation, tissue biopsy and imaging, with staging performed using the American Joint Committee on Cancer tumour–node–metastases (TNM) system.^3^

Advanced laryngeal cancers (T3 and T4) have a poor 5-year disease-specific survival of around 50 per cent.^4–6^ The decision for treatment modality is difficult and multidisciplinary teams (MDTs) may offer surgery, concurrent chemoradiotherapy or radiotherapy alone after evaluation of patient factors.^2^ Despite the clinical complexity involved, there is a lack of robust decision-making models available that can utilise the full suite of modern medical data to guide tumour-specific prognostication and treatment. There have been attempts to improve survival in this disease by examining the effect of patient demographics, risk factors, co-morbidities and pathology specimens, yet cancer survivorship has not improved.^7,8^ High volumes of imaging data, including computerised tomography (CT), magnetic resonance imaging (MRI) and positron emission tomography (PET) scans, are routinely obtained during laryngeal cancer diagnosis and staging.^2^ A detailed evaluation of radiologic information generated may be a novel avenue to refine the management of advanced laryngeal cancers.

With advances in computational techniques, it is now possible to extract quantitative information from imaging such as tumour shape, texture and intensity.^9,10^ This evolving field, which is known as radiomics, offers an objective, robust, reproducible method for imaging-data interpretation and can capture features invisible to the human eye (such as tumour heterogeneity or the microenvironment). The standard workflow involves image acquisition, tumour segmentation, feature extraction and data analysis to create a predictive model. These models have been explored in a range of cancers. Promising applications include the accurate prediction of pulmonary nodule status,^11^ treatment response and survival in lung and oesophageal cancers.^12–14^

In laryngeal cancer, radiomic models can be used to more accurately stage disease and predict survival.^15–17^ However, there is a current lack of evidence on the prognostic potential of radiomic features in advanced laryngeal cancers,^18^ especially as the choice of treatment modality remains contentious and survival rates remain poor. Our recent systematic review emphasised the potential of radiomics for improving multiple aspects of laryngeal cancer care;^18^ however, the heterogenous cohorts, poorly designed studies, and lack of data on laryngeal cancer exclusively inhibits firm conclusions. This pilot study aims to address these limitations by determining whether radiomics analysis of advanced laryngeal cancers is associated with survival, and to identify individual contrast-enhanced CT radiomic features that may act as prognostic biomarkers.

## Methods

### Study cohort

**Figure 1.**
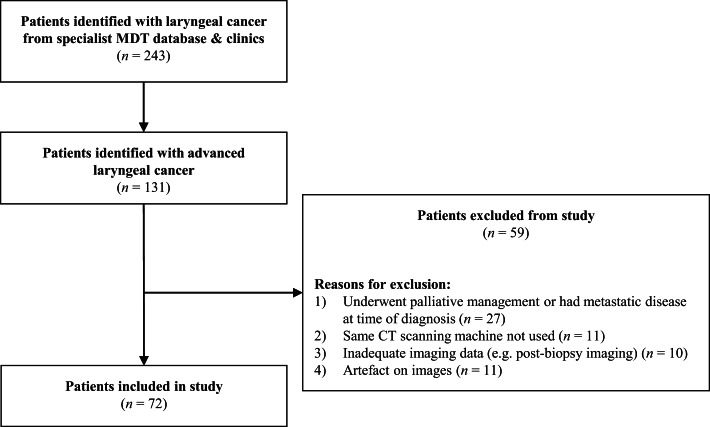
Flowchart demonstrating patients included in the study: This flowchart illustrates the patients included in the study (*n* = 72). Additional detail is provided on the number of patients excluded (*n* = 59) and the relevant rationale.

Following local trust approval, a retrospective cohort was assembled for radiomics analysis, consisting of 72 patients with advanced laryngeal (T3 and T4) squamous cell cancer (SCC) diagnosed over a 7-year period (August 2013–October 2019) at Newcastle-Upon-Tyne National Health Service Foundation Trust (a large tertiary head and neck centre). Data were sourced from otorhinolaryngology clinics or head and neck cancer MDT meetings. A set of strict selection criteria was applied (Figure 1). All patients included in the study underwent pre-biopsy, contrast-enhanced CT imaging, subsequent biopsy, and staging via TNM system.^3^ Those with metastatic disease, or who received palliative care alone (no chemotherapy, radiotherapy or surgery) were excluded. CT scans were obtained via the same scanner, scan protocol and contrast-administration protocol. Images of greater than 1 mm slice thickness or presence of artefact were excluded. Follow-up was noted as either the date of the last documented clinic consultation or date of death. Patients without sufficient medical data were excluded.

### CT technique and contrast protocol

To limit analytical bias, all images in this study were obtained with the same scanner and contrast protocol. CT scans were obtained using the Siemens SOMATOM Definition AS (Siemens AG, Munich, Germany) scanner. The contrast protocol involved 140 ml of Omnipaque™ iodinated contrast (GE HealthCare, Chicago, IL USA), which contained 300 mg of iodine per ml. The first bolus contained 70 ml of contrast. After a waiting period of 65 seconds, the chest and liver were scanned. Patients were then repositioned to have their arms to their side and asked to breathe gently throughout, before a second contrast bolus of 70 ml was injected. After another 45 seconds, the neck was scanned. The neck images were reconstructed at 1- and 3-mm-thick axial slices using the soft tissue algorithm.

### Image preparation

**Figure 2.**
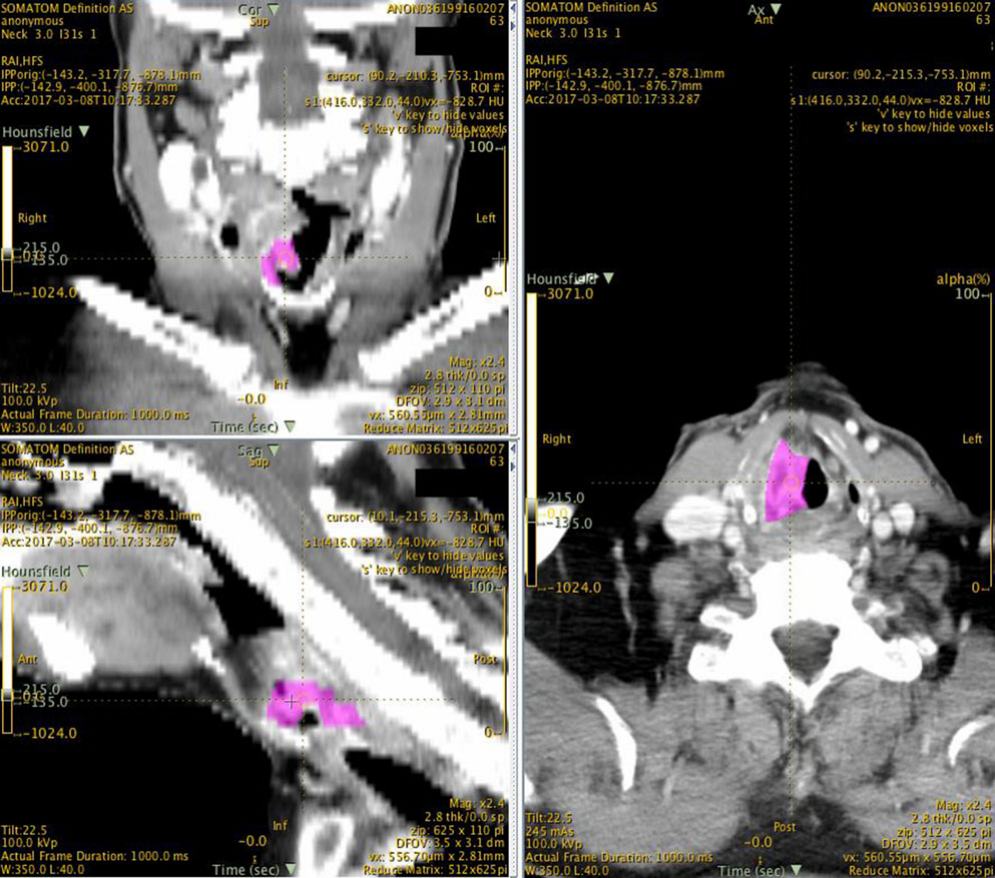
Delineation of the region of interest on the LIFEx platform: This figure illustrates axial, coronal and sagittal views of a CT scan from a patient with laryngeal cancer on the LIFEx software platform. Two blinded radiologists outlined the tumour (highlighted in pink) on each individual CT slice to obtain 3D segmentation including the whole tumor volume. This area (region of interest or volume of interest) would undergo radiomic analysis.

CT scans were prepared within the Java-based freeware LIFEx (Orsay, France; www.lifexsoft.org). LIFEx is a bespoke radiomics freeware tool which supports three-dimensional image viewing, manual tumour segmentation and subsequent texture extraction for downstream analysis.^19^ Two consultant radiologists (AM and KK) with sub-specialty interest in head and neck imaging were recruited to perform tumour segmentation in a blinded manner. Segmentation requires manually outlining the primary tumour borders across all three planes of a CT scan, marking the region of interest (Figure 2). Radiomic features (*n* = 68) were subsequently extracted from the region of interest for downstream analysis. The textural radiomic features extracted included first- and second-order features (such as intensity, grey level co-occurrence matrix, neighbourhood grey-level difference matrix, grey-level run length matrix and grey-level zone length matrix). Further details regarding individual radiomic markers can be found within the LIFEx user guide.^20^

### Statistical analysis

**Figure 3.**
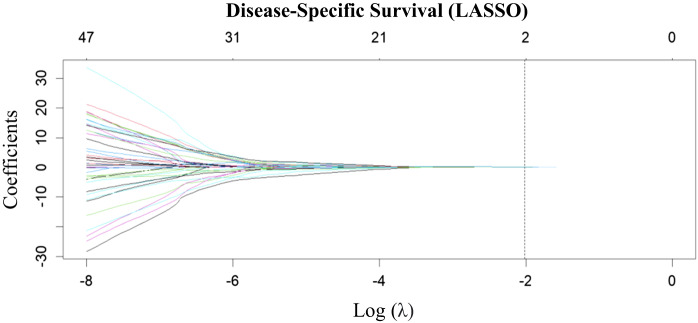
Radiomics texture feature selection using LASSO Cox regression: The ten-fold cross-validation method was used to select the model tuning parameter (λ). The log of λ was plotted against partial likelihood deviance, where a lower value suggested better model performance. The selected λ was used for co-efficient shrinkage and is depicted by the dotted line. In this figure, each individual line represents a radiomic marker. As λ increases only two radiomic markers remain (shape compacity and grey-level zone length matrix grey-level non-uniformity). LASSO = least absolute shrinkage and selection operator.

To select the best performing radiomic features for disease-specific survival analysis, a least absolute shrinkage and selection operator-based Cox regression model was employed.^17^ First, the ten-fold cross-validation method was used to select the model tuning parameter (λ). The log of λ was plotted against partial likelihood deviance, where a lower partial likelihood deviance suggested better model performance. The selected λ was used for co-efficient shrinkage (Figure 3). Within Figure 3 each line represents a radiomic marker. As λ increases only two radiomic markers remain. Ultimately this approach identified two radiomic features with non-zero co-efficients: shape compacity and grey-level zone length / matrix grey-level non-uniformity.

Shape compacity is a numerical value that compares the shape of the segmented tumour volume with a sphere, with a higher value reflecting greater irregularity. Grey-level zone length matrix – grey-level non-uniformity quantifies the number of zones within the region of interest with a specific grey level, in which a higher value equates to a higher number of zones with differing grey levels, therefore reflecting greater tumour heterogeneity.

The relationship between the two radiomic features and disease-specific survival was first explored via univariate Cox regression analysis, then in combination with conventional clinicopathological variables in a multivariable Cox regression model. Hazard ratios and 95 per cent confidence intervals (CI) were calculated for all survival analyses. To gauge the utility of incorporating radiomic features (versus clinicopathological alone), C-indices were calculated for the Cox models.

## Results

### Study cohort

Seventy-two patients with advanced laryngeal SCC were included in this pilot study (Table 1). Most patients were male (*n* = 53, 74 per cent), current smokers (*n* = 40, 56 per cent), with a median cohort age of 66 years (range 59–71 years). Nearly half of the cohort received concurrent chemoradiotherapy (*n* = 29, 40 per cent). The median follow-up period was 3.55 years (range 0.13–10.1) with a disease-specific mortality rate of 31 per cent.

**Table 1.** Pilot study cohort characteristics.

Characteristics	Study Cohort (*n* = 72)	
Median Age in Years (Range)	66	(59–71)
Gender		
– Male	53	(74%)
– Female	19	(26%)
Smoking Status		
– Non-Smoker	6	(8%)
– Ex-Smoker	26	(36%)
– Current-Smoker	40	(56%)
TNM Status (TNM 8)		
– T3	52	(72%)
– T4	20	(28%)
– N0	43	(60%)
– N+	29	(40%)
– M0	72	(100%)
Tumour Subsite		
– Subglottic	2	(3%)
– Transglottic/Glottic	44	(61%)
– Supraglottic	26	(36%)
Treatment Modality		
– Radiotherapy	10	(14%)
– Chemoradiotherapy	29	(40%)
– Surgery Alone	16	(22%)
– Surgery + Adjuvant Therapy	17	(24%)
Outcome		
– Median Follow-Up in Years (Range)	3.55	(0.13–10.1)
– Overall Mortality	35	(49%)
– Disease-Specific Mortality	22	(31%)
– Recurrence	20	(28%)

### Radiomics Analysis

**Figure 4.**
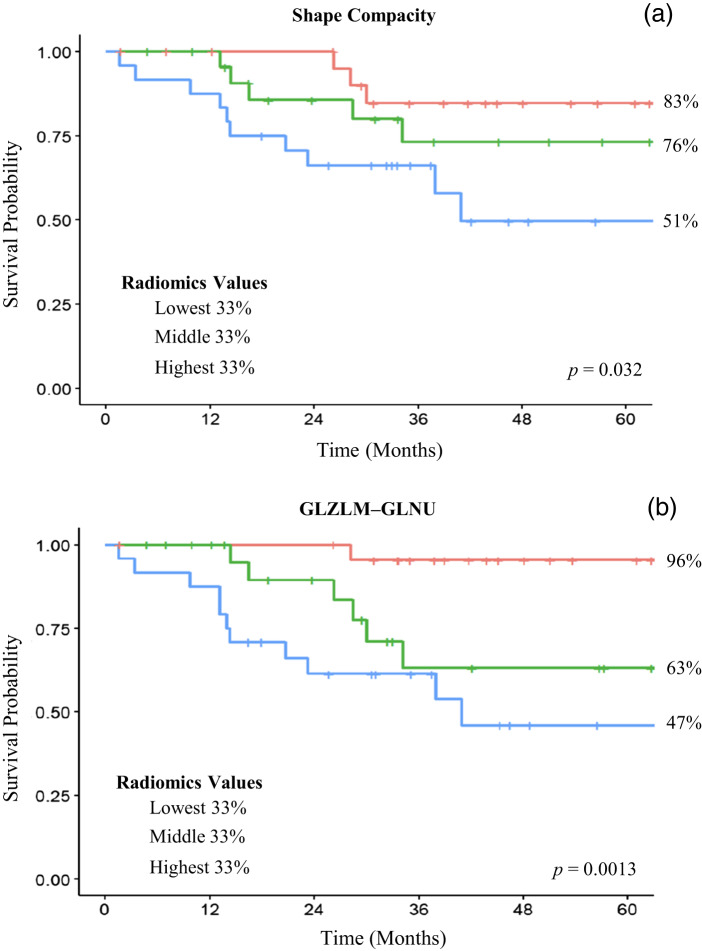
Kaplan-Meier survival curves stratified based on radiomic values: Two radiomics features were selected via the least absolute shrinkage and selection operator-based Cox regression model and assessed for prognostic significance: (a)shape compacity and (b)GLZLM-GLNU. Patients were stratified via radiomic values into upper, middle and lower terciles. The relevant Kaplan-Meier survival curves are demonstrated in this figure. GLZLM – GLNU: grey-level zone length matrix – grey-level non-uniformity

Two radiomics features were selected via the least absolute shrinkage and selection operator-based Cox regression model and assessed for prognostic significance: shape compacity and grey-level zone length matrix – grey-level non-uniformity. Independent univariate analysis of the radiomic markers also identified shape compacity and grey-level zone length matrix – grey-level non-uniformity as independent markers associated with survival. Upon univariate Cox survival analysis, a worse 5-year disease-specific survival was significantly related to greater shape compacity (hazard ratio 2.33, 95 per cent CI 1.42–3.83, *p* = 0.001) and higher grey-level zone length matrix – grey-level non-uniformity value (hazard ratio 1.69, 95 per cent CI 1.20–2.38, *p* = 0.003). The features were also stratified via radiomic values into upper, middle and lower terciles. Log-rank analysis revealed an upper tercile value in shape compacity was associated with a worse 5-year disease-specific survival when compared to the middle or lower tercile groups (51 per cent *vs* 76 per cent *vs* 83 per cent, respectively, *p* = 0.032). A similar significant pattern was observed with grey-level zone length matrix – grey-level non-uniformity (Figure 4).

When analysed together with major clinicopathological factors, the statistically significant association between worse 5-year disease-specific survival and greater shape compacity (hazard ratio 2.89, 95 per cent CI 1.40–5.93, *p* = 0.004) (Table 2A) or higher grey-level zone length matrix – grey-level non-uniformity (hazard ratio 1.64, 95 per cent CI 1.02–2.63, *p* = 0.041) remained (Table 2B). Furthermore, when both radiomic markers were combined into the same model, both a greater shape compacity (hazard ratio 2.62, 95 per cent CI 1.36–6.38, *p* = 0.024) and grey-level zone length matrix – grey-level non-uniformity (hazard ratio 1.23, 95 per cent CI 1.08–2.24, *p* = 0.040) remained statistically significant in affecting 5-year disease-specific survival (Table 2C). Of note, a Cox model utilising exclusively clinicopathological variables yielded a C-index of 0.655 (95 per cent CI 0.613–0.697) (Table 2D), versus 0.759 from the combined radiomics model (95 per cent CI 0.727–0.791) (Table 2C). The greater C-index highlights the promise of integrating radiomic features within survival modelling.

**Table 2.** Multivariable Cox regression analyses for 5-year disease-specific survival, integrating major clinicopathological variables and the selected radiomics features. Higher age and radiomics values for both shape compacity (A) and grey-level zone length matrix – grey-level non-uniformity (GLZLM – GLNU) (B) were statistically significant and associated with worse prognosis (*p* < 0.05). Furthermore, when both selected radiomics features are combined into the same model (C), higher age and radiomics values for both shape compacity and GLZLM – GLNU were statistically significant and associated with worse prognosis (*p* < 0.05). A model incorporating only clinicopathological variables (D) yielded the lowest C-index. 'Ref' refers to the comparator variable in the model. (* = *p* < 0.05).

	A	Shape Compacity		B	GLZLM – GLNU		C	Combined Model		D	Clinical-Only Model	
Variables		HR (95% CI)	*p* value		HR (95% CI)	*p* value		HR (95% CI)	*p* value		HR (95% CI)	*p* value
Age	1.06	(1.01–1.12)	0.015*	1.06	(1.01–1.12)	0.026*	1.07	(1.02–1.13)	0.016*	1.04	(1.01–1.12)	0.049*
Smoking Status												
– Non-Smoker	Ref			Ref			Ref			Ref		
– Ex-Smoker	0.16	(0.02–1.22)	0.08	0.21	(0.03–1.44)	0.11	0.31	(0.03–2.59)	0.29	0.25	(0.04–1.81)	0.17
– Current-Smoker	0.32	(0.04–2.65)	0.29	0.39	(0.05–2.98)	0.37	0.16	(0.03–2.78)	0.09	0.45	(0.06–3.34)	0.44
Tumour Stage (TNM 8)												
– T3	Ref			Ref			Ref			Ref		
– T4	0.69	(0.18–2.58)	0.58	0.84	(0.24–2.97)	0.78	0.74	(0.18–2.78)	0.62	0.78	(0.22–2.76)	0.70
Nodal Status (TNM 8)												
– N0	Ref			Ref			Ref			Ref		
– N+	2.83	(0.84–9.47)	0.09	3.28	(0.881–12.2)	0.07	2.78	(0.841–9.23)	0.10	5.07	(1.50–17.39)	0.039*
Treatment Modality												
– Surgery Alone	Ref			Ref			Ref			Ref		
– Radiotherapy	0.85	(0.17–4.32)	0.84	0.40	(0.08–2.12)	0.28	0.74	(0.08–2.12)	0.28	0.55	(0.11–2.71)	0.46
– Chemoradiotherapy	0.68	(0.14–3.31)	0.63	0.45	(0.09–2.12)	0.31	0.66	(0.09–2.12)	0.31	0.28	(0.07–1.17)	0.08
– Surgery + Adjuvant Therapy	0.48	(0.12–1.98)	0.31	0.45	(0.11–1.82)	0.26	0.47	(0.12–1.92)	0.28	0.43	(0.11–1.79)	0.25
Radiomics Feature												
– Shape Compacity	2.89	(1.40–5.93)	0.004*	—	—	—	2.62	(1.36–6.38)	0.024*	—	—	—
– GLZLM – GLNU	—	—	—	1.64	(1.02–2.63)	0.041*	1.23	(1.08–2.24)	0.040*	—	—	—
C-Index	0.737 (0.695–0.779)			0.740 (0.700–0.780)			0.759 (0.727–0.791)			0.655 (0.613–0.697)		

## Discussion

Radiomics models previously have been applied in laryngeal cancer to more accurately stage and determine thyroid cartilage invasion,^15,16^ or to predict overall survival using a combined model including clinicopathological variables.^17^ This is the first study focusing exclusively on disease-specific survival in advanced laryngeal cancer.

This pilot study used pre-biopsy, contrast-enhanced CT scans to identify two radiomic features that carry significant prognostic potential: higher values in either shape compacity (increased irregularity in tumour volume) and grey-level zone length matrix – grey-level non-uniformity (increased tumour heterogeneity) were associated with worse 5-year disease-specific survival. In our combined survival model with radiomic features, the hazard ratios suggest that rises in grey-level zone length matrix – grey-level non-uniformity or shape compacity result in a 1.23- and 2.62-times greater risk of mortality, respectively (Table 2C). Importantly, the multivariate analysis implies that these two radiomic features may be independent predictors for survival and oncological outcome.

The existing gold-standard for predicting survival in laryngeal cancer relies exclusively on clinicopathological variables, in particular the TNM staging system. This study found the inclusion of radiomics features within survival models to result in more accurate prognostication, as demonstrated by a higher C-index (0.759 *vs* 0.655) (Table 2). Incorporating both shape compacity and grey-level zone length matrix – grey-level non-uniformity into a survival model offered better discrimination versus either feature alone, emphasising the value of combining multiple radiomic features into a signature of model development. This approach has been shown previously to enhance prediction.^17^

The two radiomic features of significance in this study are grey-level zone length matrix – grey-level non-uniformity and shape compacity, both markers of tumour heterogeneity and irregularity. Tumour heterogeneity has long been recognised as an important determinant of clinical outcome. This is particularly important as laryngeal cancers are genetically and spatially heterogenous.^21–23^ Theories suggest different tumour subpopulations may respond differently to the same therapy, leading to variations in treatment response. Heterogeneity also can promote the emergence of drug-resistant clones through selection, making long-term treatment success more elusive. At present in laryngeal cancer, evaluation of tumour heterogeneity requires an invasive biopsy to obtain a tissue sample for laboratory assessment. Therefore, a non-invasive radiomic approach of determining heterogeneity (through features such as grey-level zone length matrix – grey-level non-uniformity and shape compacity) could truly enhance a patient's journey and ultimately, treatment of their cancer.^24^ In the future, imaging analysis of this nature could even limit the need for patients with extensive comorbidities to undergo general anaesthesia for invasive laryngeal biopsies.

Radiomic models may also play a future role in assisting radiotherapy and surgical planning. For example, Guo *et al*. (2020) reported CT-based radiomic features that can improve the accuracy of predicting thyroid cartilage invasion in laryngeal cancer: an important distinction indicating T4 disease that may necessitate a radical laryngectomy.^15^ Therefore, radiomics could aid surgical planning by providing the surgeon with additional information regarding the extent of resection required. With regards to assisting radiotherapy, a tumour's anatomical structure, extent of heterogeneity and corresponding radiomic features are likely to change over time during treatment. The serial analysis of radiomic features (“delta-radiomics”) over a treatment course has shown promising ability in terms of predicting disease response, radiotherapy side-effects and the need for further intervention or alteration of the treatment regime.^25,26^

Previous radiomic studies in head and neck squamous cell cancers have been limited by heterogenous cohorts with non-uniform scanning protocols and equipment.^18^ To increase the quality of radiomics analysis in this pilot, careful exclusion criteria were applied to obtain maximally uniform CT scans across a well-defined cohort of patients. In addition, image segmentation was performed by blinded specialist head and neck radiology consultants to account for interobserver variability and reduce human error. Finally, because LIFEx is a freely available, internationally validated radiomics software package,^19^ other institutions will be able to apply validated risk-prediction models and perform consistent, multi-centre research easily without incurring additional cost.

- Advanced laryngeal cancers have poor outcomes and are challenging to manage
- Radiomics describes the quantitative analysis and extraction of features from medical imaging
- These radiomic features may capture characteristics such as tumour volume and heterogeneity
- In this retrospective pilot study, two CT radiomic features are identified as prognostic biomarkers for disease-specific survival
- Integrating radiomics into prognostic models may refine and guide tumour-specific management

The primary limitations of this pilot study include its retrospective, single-centre nature, and small sample size. However, the promising results demonstrate proof-of-concept and highlight the urgent necessity for a large, multi-centred prospective study in the future. The purpose of this pilot study was not to create a predictive model but identify radiomic markers that can be used to guide future work. A more robust analysis may identify clinically significant radiomic models that can advance the care of patients with laryngeal cancer.

## Conclusion

In this pilot, two radiomic features were identified as significant prognostic biomarkers for disease-specific survival in advanced laryngeal cancer. The study further highlights that models integrating radiomic features are superior to models composed solely of clinicopathological factors in advanced laryngeal cancer. In the future, this will allow the development of clinically relevant radiomics-based models that can refine the management of advanced laryngeal cancer and in turn improve survivorship.
